# Performance of the ROX index to predict intubation in immunocompromised patients receiving high-flow nasal cannula for acute respiratory failure

**DOI:** 10.1186/s13613-021-00801-z

**Published:** 2021-01-27

**Authors:** Virginie Lemiale, Guillaume Dumas, Alexandre Demoule, Frederic Pène, Achille Kouatchet, Magali Bisbal, Saad Nseir, Laurent Argaud, Loay Kontar, Kada Klouche, Francois Barbier, Amelie Seguin, Guillaume Louis, Jean-Michel Constantin, Julien Mayaux, Florent Wallet, Vincent Peigne, Christophe Girault, Johanna Oziel, Martine Nyunga, Nicolas Terzi, Lila Bouadma, Alexandre Lautrette, Naike Bige, Jean-Herle Raphalen, Laurent Papazian, Fabrice Bruneel, Christine Lebert, Dominique Benoit, Anne-Pascale Meert, Samir Jaber, Djamel Mokart, Michael Darmon, Elie Azoulay, The Groupe de Recherche en Reanimation Respiratoire du patient d’Onco-Hématologie (GRRR-OH)

**Affiliations:** 1 AP-HP, Hôpital Saint-Louis, Medical Intensive Care Unit and Department of Biostatistics, APHP, Hopital St-Louis, 1 avenue Claude Vellefaux, 75010 Paris, France; 2 Medical Intensive Care Unit and Respiratory Division, APHP, Hopital Pitie-Salpetriere, Sorbonne University, Paris, France; 3 Medical Intensive Care Unit, Hospital Cochin, APHP, Universite Paris Descartes, Paris, France; 4 Medical Intensive Care Unit, CHRU, Angers, France; 5 Intensive Care Unit, Paoli Calmettes Institute, Marseille, France; 6 Critical Care Center, CHU de Lille, Lille, France; 7 Medical Intensive Care Unit, Hospices Civils de Lyon, Hopital Edouard Herriot, Lyon, France; 8 Medical Intensive Care Unit, INSERM U1088, Amiens University Hospital, Amiens, France; 9 Medical Intensive Care Unit, CHU de Montpellier, Montpellier, France; 10 Medical Intensive Care Unit, La Source Hospital, CHR Orleans, Orleans, France; 11 Medical Intensive Care Unit, Hotel Dieu, CHU de Nantes, Nantes, France; 12 Intensive Care Unit, CHR de Metz-Thionville, Metz, France; 13 Department of Perioperative Medicine, CHU Clermont-Ferrand, Clermont-Ferrand, France; 14 Intensive Care Unit, Lyon Sud Medical Center, Lyon, France; 15 Intensive Care Unit, Centre Hospitalier Metropole-Savoie, Chambery, France; 16 Medical Intensive Care Unit, Hospital Charles Nicolle, Rouen, France; 17 Medical Intensive Care Unit, Avicenne University Hospital, Bobigny, France; 18 Intensive Care Unit, Roubaix Hospital, Roubaix, France; 19 Medical Intensive Care Unit, CHU de Grenoble Alpes, Grenoble, France; 20 Medical Intensive Care Unit, CHU Bichat, Paris, France; 21 Medical Intensive Care Unit, Gabriel-Montpied University Hospital, Clermont-Ferrand, France; 22 Medical Intensive Care Unit, CHU St-Antoine, Paris, France; 23 Department of Anesthesia and Critical Care, Necker Hospital, Paris, France; 24 Reanimation Des Detresses Respiratoires Et Infections Severes, Assistance Publique—Hopitaux de Marseille, Hopital Nord, Faculte de Medecine, Aix-Marseille Universite, Marseille, France; 25 Medical Intensive Care Unit, Andre Mignot Hospital, Versailles, France; 26 Intensive Care Unit, Centre Hospitalier Departemental Les Oudairies, La Roche Sur Yon, France; 27 Department of Intensive Care, Ghent University Hospital, Ghent, Belgium; 28 Service de Médecine Interne, Soins Intensifs & Urgences Oncologiques, Institut Jules Bordet, Bruxelles, Université Libre de Bruxelles (ULB), Brussels, Belgium; 29 Montpellier University Hospital, PhyMedExp, INSERM U-1046, CNRS, 34295 Montpellier, France

**Keywords:** High-flow nasal oxygen, Immunocompromised, Acute respiratory failure

## Abstract

**Background:**

Delayed intubation is associated with high mortality. There is a lack of objective criteria to decide the time of intubation. We assessed a recently described combined oxygenation index (ROX index) to predict intubation in immunocompromised patients. The study is a secondary analysis of randomized trials in immunocompromised patients, including all patients who received high-flow nasal cannula (HFNC). The first objective was to evaluate the accuracy of the ROX index to predict intubation for patients with acute respiratory failure.

**Results:**

In the study, 302 patients received HFNC. Acute respiratory failure was mostly related to pneumonia (*n* = 150, 49.7%). Within 2 (1–3) days, 115 (38.1%) patients were intubated. The ICU mortality rate was 27.4% (*n* = 83). At 6 h, the ROX index was lower for patients who needed intubation compared with those who did not [4.79 (3.69–7.01) vs. 6.10 (4.48–8.68), *p* < 0.001]. The accuracy of the ROX index to predict intubation was poor [AUC = 0.623 (0.557–0.689)], with low performance using the threshold previously found (4.88). In multivariate analysis, a higher ROX index was still independently associated with a lower intubation rate (OR = 0.89 [0.82–0.96], *p* = 0.04).

**Conclusion:**

A ROX index greater than 4.88 appears to have a poor ability to predict intubation in immunocompromised patients with acute respiratory failure, although it remains highly associated with the risk of intubation and may be useful to stratify such risk in future studies.

## Background

In immunocompromised patients with acute respiratory failure (ARF), invasive mechanical ventilation remains associated with a high mortality rate [1]. Several oxygenation strategies to avoid intubation have been tested in this setting. More recently, high-flow nasal cannula (HFNC) has been evaluated in non-immunocompromised patients, resulting in an improved oxygenation ratio and reduced intubation rate, as shown in several recent meta-analyses [2, 3]. In immunocompromised patients, the role of HFNC seems less clear, and a higher oxygenation rate does not translate into improved outcomes [4, 5]. Moreover, all these studies highlight the risk of delayed intubation, which is associated with a higher mortality rate [6]. Therefore, particular attention should be paid to the time of intubation to determine which patient would benefit from a non-invasive strategy of oxygenation [7]. So far, there is a lack of objective criteria to decide intubation, particularly within the first hours after the onset of the oxygenation strategy. Some recent studies have assessed several indices, including clinical and respiratory parameters [8, 9], such as the ROX index—including oxygen saturation (SpO_2_), fraction of inspired oxygen (FiO_2_), and respiratory rate (RR)—which is easy to use [10, 11]. A ROX index (SpO_2_/FiO_2_/RR) over 4.88 within 2–12 h of starting HFNC was associated with a lower risk of intubation. The area under the curve (AUC) of the receiver operating characteristic (ROC) curve in the validation cohort was 0.703 (0.616–0.790) at 6 h and 0.752 (0.664–0.840) at 12 h. However, the studies were carried out in non-immunocompromised patients with pneumonia-related ARF. For immunocompromised patients, ARF aetiologies are numerous and response to HFNC could vary [7].

The aim of this study was to validate the ROX index in an external dataset, including only immunocompromised patients with ARF.

## Methods

This is an ancillary study including two previous trials, IVNICTUS and HIGH [5, 12]. The IVNICTUS study, described elsewhere, evaluated an oxygenation strategy using non-invasive ventilation (NIV) versus oxygen in immunocompromised patients with ARF [12]. Oxygen could be maintained by HFNC in both groups. The HIGH study, also described elsewhere, compared oxygen versus high-flow nasal oxygen in immunocompromised patients with ARF [5]. In both studies, HFNC was used according to recommendations (gas flow of 50–60 L/min, FiO_2_ adjusted for SpO_2_ over 92%, temperature 37 °C). Institutional review board agreement and written informed consent were obtained from each patient or surrogate decision-maker (CPP Ile de France IV 2012/11SC and IDRCB: 2016-A00220-51). The inclusion criteria in both studies were ARF, defined by tachypnea over 30/min, respiratory distress (laboured breathing) and SpO_2_ < 90% on room air at ICU admission. The non-inclusion criteria were ARF being related to isolated cardiogenic pulmonary oedema and invasive mechanical ventilation at ICU admission. Persistent hypoxemia or dyspnoea were among the intubation criteria.

The secondary analysis included all patients who received HFNC at least 6 h after randomization. The exclusion criteria for this secondary analysis were: do not intubate order, intubation before 6 h, and missed data on the ROX index at 6 h.

The ROX index (SpO_2_/FiO_2_)/RR) was calculated at 6 h and also at 12 h for patients included from the IVNICTUS study (the variable was not available in the HIGH study). Patient characteristics at ICU admission and outcomes were also recorded.

The ARF aetiology was determined using the appropriate diagnostic strategy and was classified as bacterial or viral pneumonia, opportunistic infection, lung involvement by the underlying disease, drug-related pulmonary toxicity, other identified cause, or undetermined aetiology. The diagnostic strategy and criteria for each diagnosis are described elsewhere [13]. Patients with ARF related to isolated cardiogenic oedema were not included in the trials [5, 12]. Radiographic patterns at ICU admission were described as 1–2 or 3–4 involved quadrants. The primary endpoint was the need for intubation throughout the ICU stay. The primary objective was to assess the ability to discriminate between patients who would need intubation and patients who would not require intubation.

The secondary objective was to evaluate the ability of the ROX index to stratify intubation risk.

### Statistical analyses

Results were expressed as median and 25th and 75th quartiles (Q1–Q3) for the quantitative data, and numbers and percentages for the categorical data. The quantitative variables were compared using the Student’s *t* test, or the Wilcoxon test in case of non-normal distribution. The qualitative variables were compared using the Chi-square test or Fisher’s exact test, as appropriate. Missing data were not imputed, and the number of analysed patients is described in each table in the results section.

Several analyses were carried out. The first was a univariate analysis to describe the characteristics and outcomes of intubated and non-intubated patients, while the second analysis assessed the predicting value of the ROX index for intubation using a ROC curve to discriminate between patients who needed intubation and those who had HFNC success. The thresholds found in a previous study were then evaluated for sensibility, specificity, likelihood positive ratio and likelihood negative ratio [10, 11].

In a third analysis, the ROX index was adjusted with other variables associated with a high risk of intubation [14] that could be assessed during the first 24 h of ICU. A multivariate logistic model was performed including characteristics at randomization (oxygen flow over 9 L/min, study), ARF related to pneumonia and SOFA score at day 1. All clinically relevant variables decided a priori and those with a low level of missing data were included in the multivariate analysis, even in cases of non-significant difference in the univariate analysis. The odds ratios (ORs) of variables present in the final model are given with their 95% confidence intervals (CI).

### Several sensitivity analyses were performed

Firstly, a ROC curve analysis was performed to assess the accuracy of the multivariate model to discriminate between patients who would need intubation and those who would not. Secondly, in order to assess the impact of the ROX index, we assessed the predicting value of a modified multivariate model of intubation risk, excluding the ROX index, using a ROC curve to discriminate between patients who needed intubation and those who had HFNC success.

Thirdly, the risk stratification of the ROX index was then explored with the probability of intubation for each quartile of the ROX index.

Fourthly, one sensibility analysis was carried out excluding all patients who received NIV. In the INVICTUS study [12], half of the patients were randomized to receive a NIV session immediately after randomization. Although the ROX index was calculated outside a NIV session, the NIV session may modified the risk of intubation, the RR and the SpO_2_ at hour 6. To assess the impact of this device, we performed the same analysis excluding patients who received NIV.

The ROX index at 12 h was also analysed, where available (in the IVNICTUS cohort only).

All analyses were carried out using the R 3.3.3 statistical software, and the statistical significance level was fixed at 0.05.

## Results

**Fig. 1:**
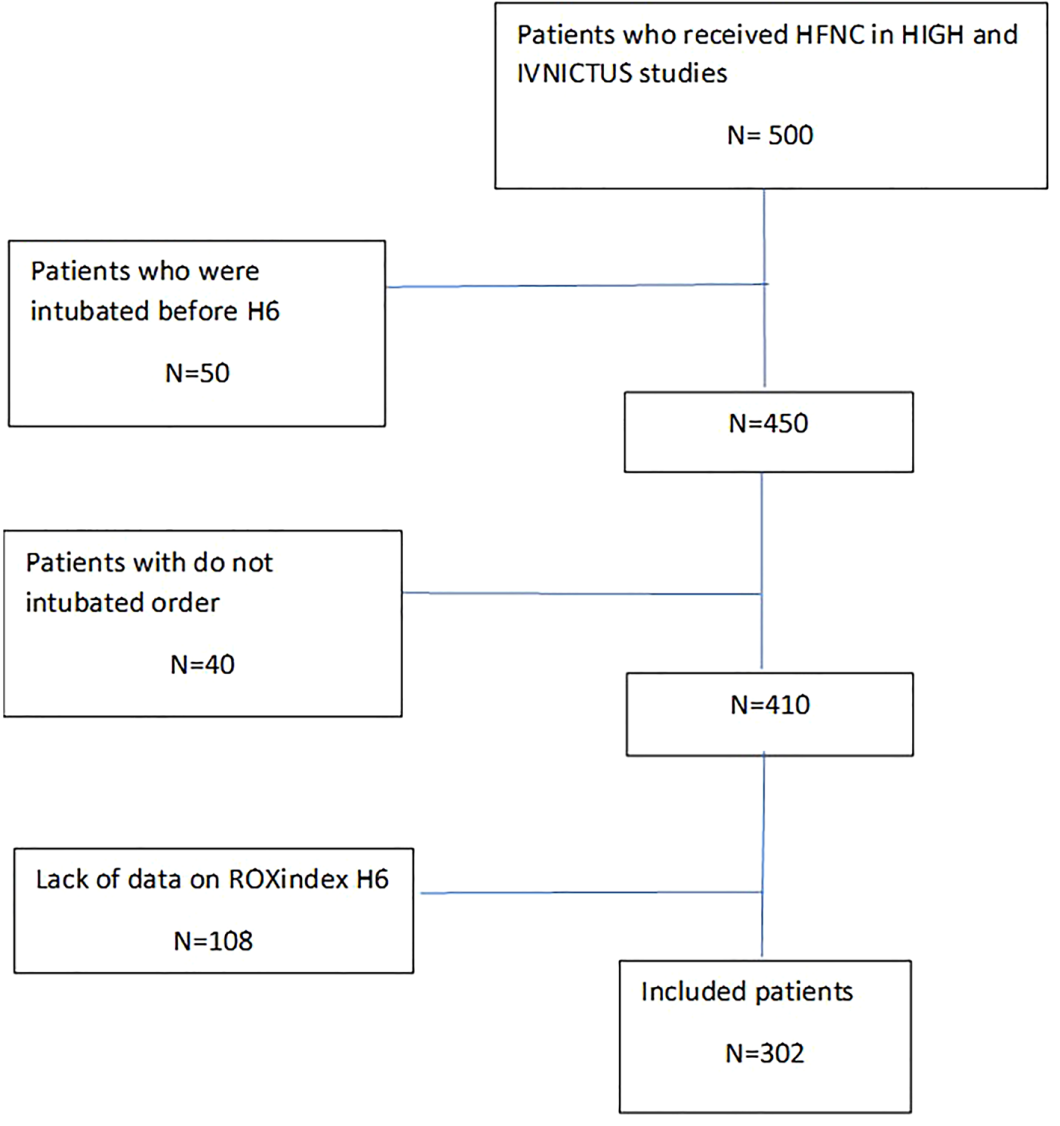
Flowchart

This study included 302 patients admitted with ARF (Fig. 1). Patients were mostly male (*n* = 208, 68.9%), with a median age of 63 (55–70). The underlying disease was mostly haematological malignancy (*n* = 225, 74.5%), while ARF was related to viral or bacterial pneumonia (*n* = 150, 49.7%), opportunistic pneumonia (*n* = 54, 17.9%), toxicity or underlying disease (*n* = 26, 8.6%) and other miscellaneous reasons (*n* = 52, 17.2%) including pulmonary embolism, extrapulmonary acute respiratory distress syndrome (ARDS), pleural effusion and atelectasis.

The oxygen flow needed at randomization was 10 (7–15) L/min, and 166 (55%) patients required an oxygen flow over 9 L/min. Forty-five (14.9%) patients received NIV and HFNC. The RR was 27 (22–33)/min, 30 min after randomization. In the HIGH study, there were guidelines for HFNC use with at least 50 L/min [5]. In the IVNICTUS study, HFNC flow was 50 (35–50) L/min at initiation. For 122 (49.6%) patients, 3–4 quadrants were involved on the chest X-ray. The median ROX index at 6 h was 5.62 (4.17–8.29).

**Fig. 2:**
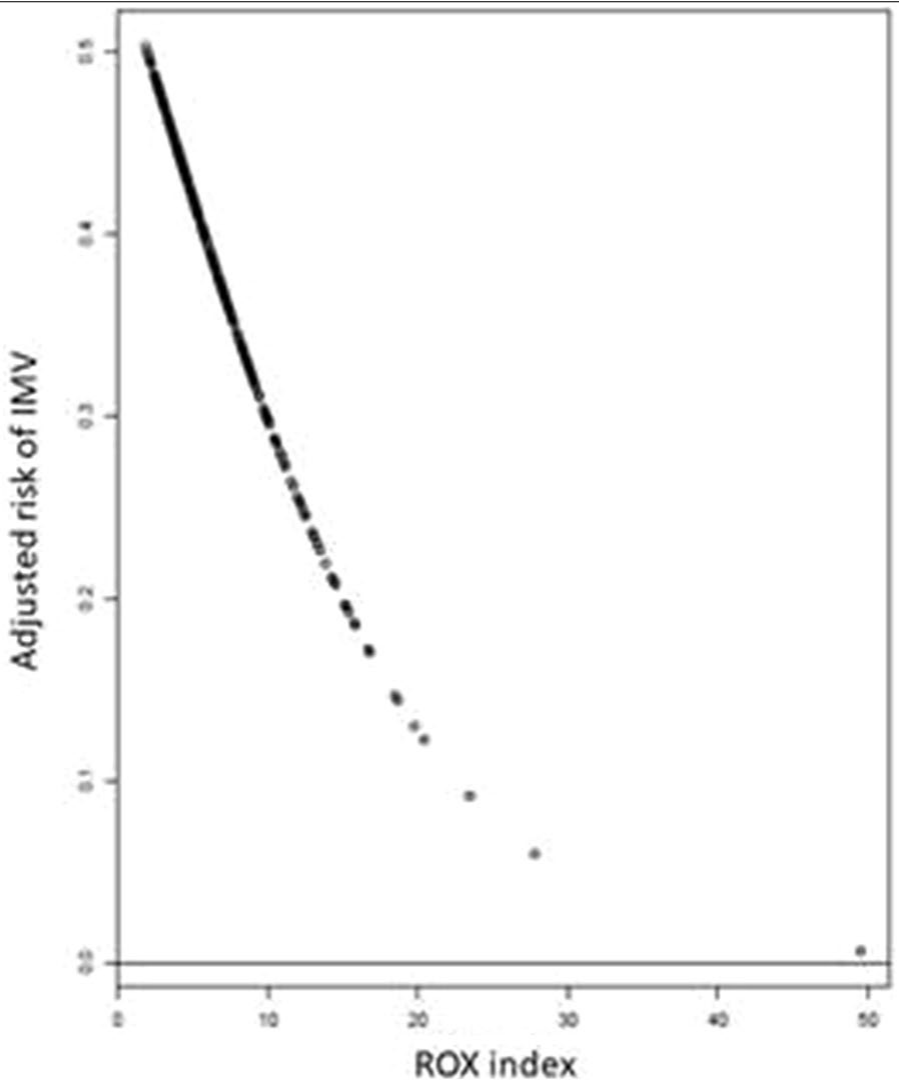
Probability of intubation according the ROX index

Throughout the ICU stay, invasive mechanical ventilation was required by 115 (38.1%) patients within the 2 (1–3) days. The length of hospital stay was 24 (14–29) days, while the ICU mortality rate was 27.4% (*n* = 83). Table 1 summarizes patient characteristics at ICU admission and outcomes based on the need for invasive mechanical ventilation. The ROX index at 6 h was significantly different between patients who required intubation and those who did not. The probability of intubation increased when the ROX index decreased (Fig. 2). Table 2 describes patient characteristics according to the ROX index value (over or below 4.88).

**Table 1:** Characteristics and outcomes based on the need for intubation

Variables	Patients who need intubation (*n* = 115)	Patients who were not intubated (*n* = 187)	*p*
Age (year) median, (IQR)	63 (53–69)	63 (56–71)	0.25
Gender, male (*n*, %)	81 (70)	127 (68)	0.74
Comorbidities (*n*, %)			
Cardiovascular	59 (58)	92 (56)	0.85
Pulmonary	41 (40.6)	63 (38.7)	0.85
Kidney	15 (15)	34 (21)	0.31
Charlson Index median, (IQR) missing data = 36	4 (3–6)	5 (4–7)	0.006
Underlying disease (*n*, %)			
Haematological malignancy, *n* (%)	90 (78)	135 (72)	0.12
Allogeneic stem cell transplant, *n* (%)	15 (14.7)	13 (9.3)	0.27
Performance status < 2 (*n*, %)	68 (59)	111 (59)	0.99
Number of quadrant involved = 3 or 4 (*n*, %) missing data = 56	56/96 (44)	66/150 (31)	0.04
Aetiology of ARF (*n*, %)			0.07
Bacterial or viral pneumonia	58 (50)	92 (49.5)	
Opportunistic infection	27 (23.5)	27 (14.4)	
Lung involvement by the underlying disease drug-related pulmonary toxicity	7 (6)	19 (10)	
Other identified causes	13 (11)	39 (21)	
No identified cause	9 (7.8)	8 (4.3)	
Neutropenia recovery at admission (*n*, %)	15 (13)	13 (7)	0.12
SOFA day 1 missing data = 25	6 [3–9]	4 [2–6]	< 0.001
RR 30 min after randomization median, (IQR) missing data = 14	29 (22.5–35)	26 (22–32)	0.02
Oxygen flow > 9 L/min at randomization, *n* (%)	66 (57.4)	100 (53.5)	0.02
ROX index at H6	4.79 [3.69–7.01]	6.10 [4.48–8.68]	< 0.001
NIV group	20 (17.4)	25 (13.3)	0.43
Glasgow score at 15 at Day 1 (n,%)	97 (84)	160 (86)	0.86
Length between ICU admission and intubation	2 (1–3)	NA	NA
Outcomes			
Vasopressor during ICU stay, *n* (%)	99 (86)	34 (18)	< 0.001
Renal replacement therapy during ICU stay, *n* (%)	38 (33)	11 (6)	< 0.001
End of life decision during ICU stay, *n* (%)	31 (29)	10 (5.4)	< 0.001
ICU mortality (*n*,%)	64 (55.7)	19 (10.2)	< 0.001
Length of ICU stay for survival patient (*n* = 219)	17 (12.5–27)	7 (5–11)	< 0.001
Length of hospital stay median, (IQR) (*n* = 302)	32 (15–60)	21 (13–32)	0.001

ARF: acute respiratory failure, NIV: non-invasive ventilation, RR: respiratory rate

**Table 2:** Characteristics and outcomes based on ROX index over or under 4.88

Variables	Patients with ROX index H6 > 4.88 (*n* = 184)	Patients with ROX index H6 ≤ 4.88 (*n* = 118)	*p*
Age (year) median, (IQR)	63.5 (55–71)	63 (54–69)	0.57
Gender, male (*n*, %)	130 (71)	78 (66)	0.48
Comorbidities (*n*, %)			
Cardiovascular	93 (58)	58 (56)	0.8
Pulmonary	61 (38)	43 (41)	0.69
Renal	28 (18)	21 (20)	0.72
Charlson Index median, (IQR) missing data = 36	5 (4–7)	5 (3–6)	0.42
Underlying disease (*n*, %)			
Haematological malignancy, *n* (%)	139 (75)	86 (73)	0.36
Allogeneic stem cell transplant, *n* (%)	19 (11)	9 (8)	0.56
Performance status > 2 (*n*, %)	63 (34)	42 (36)	0.13
Number of quadrant involved = 3 or 4 (n,%) missing data = 56	68 (45)	54 (57)	0.09
Aetiology of ARF (*n*, %)			0.84
Bacterial or viral pneumonia	92 (50)	58 (49)	
Opportunistic infection	29 (16)	25 (21)	
Lung involvement by the underlying disease Drug-related pulmonary toxicity	16 (9)	10 (8)	
Other identified causes	33 (18)	19 (16)	
No identified cause	12 (6)	5 (4)	
Neutropenia recovery at admission (n,%)	18 (10)	7 (6)	0.33
SOFA day 1 missing data = 25	4 (2–7)	4.5 (2–7)	0.94
RR 30 min after randomization median, (IQR) missing data = 14	25 (21–30)	31 (25–35)	< 0.001
Oxygen flow > 9 L/min at randomization, *n* (%)	94 (51)	72 (13)	0.49
NIV group	30 (16)	15 (13)	0.45
Glasgow score at 15 at Day 1 (*n*, %)	160 (87)	97 (82)	0.52
Outcomes			
Intubated during ICU (*n*, %)	55 (30)	60 (51)	< 0.001
Length between ICU admission and intubation	3 (1–4.5)	2 (1–2.5)	0.005
Vasopressor during ICU stay, *n* (%)	71 (39)	62 (52)	0.03
Renal Replacement Therapy during ICU stay, *n* (%)	30 (16)	19 (16)	0.32
End of life decision during ICU stay, *n* (%)	47 (26)	47 (36)	0.02
Day 28 mortality (*n*,%)	39 (21)	44 (37)	0.003
Length of ICU stay, median (IQR) survival patient (*n* = 219)	8 (5–13)	10 (6–17.7)	0.02
Length of hospital stay median, (IQR) (*n* = 302	24 (15–38)	22 (12–40)	0.52

### Prediction of intubation need using the ROX index at 6 h

Additional file 1: Figure S1 describes the ROC curve for the probability of intubation based on the ROX index in our cohort. The AUC was 0.623 (0.557–0.689). Using the threshold previously found (ROX index over 4.88 at 6 h, was associated with lower intubation rate) [11], the prediction of intubation was very low (sensibility of 52.1%, specificity of 68.9%, positive likelihood ratio of 1.69 and negative likelihood ratio of 0.69). Also, using an ROX index below 3.47 [11], the prediction of HFNC failure had higher sensibility (90.9%) but a specificity of 21%, a positive predictive value of 65%, a negative predictive value of 59%, a positive likelihood ratio of 2.3 and a negative likelihood ratio of 0.86.

### Adjusted ROX index at 6 h based on other risks of intubation

In a multivariate analysis that included patients’ characteristics at admission (Table 3), a higher ROX index was still independently associated with a lower intubation rate (OR = 0.89 [0.82–0.96], *p* = 0.04). The SOFA score at day 1 was associated with a higher risk of intubation. There was no interaction between SOFA J1 and ROX index. A modified model without SOFA day 1 did not modify the result on the ROX index [OR = 0.89 (0.83–0.96)].

**Table 3:** Adjusted ROX index 6 h after HFNC onset according other risk factors of intubation in all cohorts (multivariate analysis)

Variables	OR (95% CI)	*p*
Oxygen flow at randomization > 9 L/min	1.10 (0.64–1.88)	0.27
IVNICTUS study	1.03 (0.58–1.83)	0.29
ARF related to pneumonia	1.06 (0.63–1.76)	0.26
SOFA Day 1 (per point increase)	1.17 (1.08–1.27)	0.04
ROX H6 (per point increase)	0.89 (0.82–0.96)	0.04

Oxygen flow at ICU admission was dichotomized below or over 9 l/min

Patients form IVNICTUS study were compared to patients from HIGH study

ARF aetiology was dichotomized between ARF related to bacterial or viral pneumonia versus all other reason of ARF

SOFA Day 1 was sequential organ failure assessment

ROX index was used as continuous variable

Characteristics of the multivariate model

Hosmer and Lemeshow goodness of fit test p = 0.65

The AUC of the ROC curve of the predictive model = 0.66

The relationship between ROX index and lod(odds) of intubation was linear

*ARF* acute respiratory failure, *NIV* non-invasive ventilation, *OR* odds ratio, *CI* confidence interval

Additional file 1: Figure S2 describes the probability of intubation according to the multivariable model including the ROX index.

### Sensibility analyses

Additional file 1: Figure S3 describes the ROC curve of the probability of intubation with an adjusted ROX index at 6 h. The AUC of this curve was 0.662 (0.596–0.729). This ROC curve was compared to the one from a model including all variables a priori associated with intubation but without the ROX index at 6 h (Additional file 1: Figure S4). The AUC of this second ROC curve was 0.632 (0.563–0.702). The model including ROX index showed a better overall fit (*p* = 0.002).

Additional file 1: Figure S5 describes the risk stratification of intubation according to the quartile of the ROX index. The probability of intubation decreased with a high ROX index, without overlap between each quartile, leading to a good performance of risk stratification.

Statistical analysis of the impact of NIV was explored by excluding the patients who eventually received NIV within the first 6 h. The AUC of the ROC curve was 0.630 (0.558–0.703). In the multivariate analysis, the ROX index at 6 h was still independently associated with a lower intubation rate [OR = 0.90 (0.82–0.97), *p* = 0.01]. In this secondary analysis, the SOFA score at day 1 was also associated with intubation [OR = 1.16 (1.06–1.27), *p* = 0.001].

Finally, in the IVNICTUS study, the ROX index was available for 89 out of 95 (93.6%) patients, among whom 39 (43.8%) were intubated during the ICU stay after 12 h. The AUC of the ROC curve was 0.655 (0.538–0.772).

## Discussion

In this study, the ROX index 6 h after HFNC onset had a poor performance in discriminating between patients who would be intubated or not, but this index could be a tool for risk stratification. Also, the ROX index was still associated with the risk of intubation, even when other significant predictors were included, such as the SOFA score [14].

Nevertheless, its accuracy to discriminate between patients who would need intubation and those who would receive only a non-invasive oxygenation strategy was very low, with low specificity and sensibility. In addition, the performance of a lower threshold (3.47) to predict HFNC failure was poor. Despite the low accuracy of this index to discriminate between patients who would ultimately need intubation or not, the ROX index can be easily recorded at the bedside. However, although RR, saturation and oxygen levels could be helpful for extreme values, the performance of these parameters to determine the intubation risk remains poor for medium values. Moreover, the ROX index at 6 h is a static measurement of clinical condition in ARF patients. Predicting intubation remains difficult and may depend on several conditions. Apart from respiratory parameters, the decision to intubate would also be determined by other organ dysfunctions, particularly haemodynamic and neurological dysfunctions. Repeated measures of oxygenation may also be of importance [11]. Few studies included the neurological and haemodynamic status in their predictive score [9]. Our study included ARF aetiology, oxygen needs at randomization and organ failure in the multivariate analysis, as these parameters have been associated with a risk of intubation [14]. Regarding the duration of oxygen needs and the assumed evolution of the disease, intubation would be necessary in the most severe patients and should not be delayed [6]. The physician’s experience could also influence the decision to intubate. As not all these characteristics can be included in a sole oxygenation index, this contributes to the poor accuracy of the ROX index to discriminate between patients who would need intubation and those who would not.

Moreover, the ROX index is a single measure and may not reflect the clinical evolution of a patient. With the sensitivity analysis at 12 h, we explored a second ROX index value, but this was also only a static measurement. Mauri et al. assessed the response to different levels of gas flow [15]; interestingly, they found a subgroup of patients where the ROX index of the most severe one rose within 20 min of increasing the gas flow on the HFNC. It is possible that a higher gas flow could increase lung recruitment. These changes highlighted the difficulty of determining a sole oxygenation index to distinguish between patients who would need intubation and those who would not. Roca et al. suggested a repeated measurement of the ROX index to increase its accuracy [11]. Also, this result identified the need for a scalable variable to detect a worsening outcome. Probably only the analysis of a large dataset could determine the profile of a patient who would need intubation.

According to our study, more than accuracy to discriminate between patients who need intubation or not, the ROX index may have a good performance for risk stratification [16]. Contrary to the limited clinical application of the ROX index itself, the stratification in low, intermediate and high-risk patients may be helpful for the early identification of patients who would fail the HFNC trial in further studies. Such results should be confirmed in further studies.

This study has several limitations. Firstly, it was a retrospective analysis of data, and the reason for intubation was not described. Although intubation occurred within the first days of ICU admission, a later intubation may not be related to the ROX index value at 6 h. Moreover, 108 patients could not be included in this analysis because of missing data. Those patients might have modified the conclusion. However, the study included a high number of patients, as in previous studies on the ROX index [11].

Secondly, this external validation cohort included only immunocompromised patients. Indeed, different aetiology of ARF [13], different oxygenation needs and different comfort related to the device [17] in immunocompromised patients may have modified the response to HFNC and, consequently, the ROX index accuracy.

Thirdly, we could not analyse the need for a vasopressor or other organ failure supply before intubation. Only the SOFA score at day 1 was analysed, even in cases of intubation. This may be a major limitation, because some patients would display haemodynamic instability after intubation. However, we did not detect any interaction between the ROX index and the SOFA day 1 score. Only prospective data could provide a remedy to this point.

Fourthly, some patients received NIV within the first 6 h. Those NIV sessions could have increased lung recruitment and modified the ROX index. However, the sensibility analysis excluding those patients did not show any difference, and the ROX index remained associated with the risk of intubation.

Fifth, unlike previous studies [11], several ARF aetiologies were included in our research. In the setting of immunocompromised patients, the length of oxygen needs and the severity of ARF may vary according to the ARF aetiology. However, half of patients had bacterial pneumonia.

## Conclusion

Although the ROX index appears to have a low performance in predicting intubation among immunocompromised patients with acute respiratory failure, it still represents one of the strongest associated factors with intubation. More data including non-respiratory parameters may be included in further prospective cohorts to confirm these results. However, the index seems to have a good performance for risk stratification and may be useful for further studies.

## Supplementary Information

**Additional file 1: Figure S1.** ROC curve for ROX index 6 hours after HFNC onset. **Figure S2.** Probability of intubation according to the multivariable model including ROX index. **Figure S3.** ROC curve of adjusted ROX index 6 hours after HFNC onset. **Figure S4.** Comparison between ROC curves of adjusted ROX index and modified model without ROX index. **Figure S5.** Probability of intubation according to ROX index quartile.

## Data Availability

The datasets used and/or analysed during the current study are available from the corresponding author on reasonable request.
