# Correction to: Performance of the ROX index to predict intubation in immunocompromised patients receiving high-flow nasal cannula for acute respiratory failure

**DOI:** 10.1186/s13613-021-00894-6

**Published:** 2021-07-09

**Authors:** Virginie Lemiale, Guillaume Dumas, Alexandre Demoule, Frederic Pène, Achille Kouatchet, Magali Bisbal, Saad Nseir, Laurent Argaud, Loay Kontar, Kada Klouche, Francois Barbier, Amelie Seguin, Guillaume Louis, Jean-Michel Constantin, Julien Mayaux, Florent Wallet, Vincent Peigne, Christophe Girault, Johanna Oziel, Martine Nyunga, Nicolas Terzi, Lila Bouadma, Alexandre Lautrette, Naike Bige, Jean-Herle Raphalen, Laurent Papazian, Fabrice Bruneel, Christine Lebert, Dominique Benoit, Anne-Pascale Meert, Samir Jaber, Djamel Mokart, Michael Darmon, Elie Azoulay, The Groupe de Recherche en Reanimation Respiratoire du patient d’Onco-Hématologie (GRRR-OH)

**Affiliations:** 1 Medical Intensive Care Unit and Department of Biostatistics, APHP, Hopital St-Louis, 1 avenue Claude Vellefaux, 75010 Paris, France; 2 Medical Intensive Care Unit and Respiratory Division, APHP, Hopital Pitie-Salpetriere, Sorbonne University, Paris, France; 3 Medical Intensive Care Unit, Hopital Cochin, APHP, Universite Paris Descartes, Paris, France; 4 Medical Intensive Care Unit, CHRU, Angers, France; 5 Intensive Care Unit, Paoli Calmettes Institut, Marseille, France; 6 Critical Care Center, CHU de Lille, Lille, France; 7 Medical Intensive Care Unit, Hospices Civils de Lyon, Hopital Edouard Herriot, Lyon, France; 8 Medical Intensive Care Unit, INSERM U1088, Amiens University Hospital, Amiens, France; 9 Medical Intensive Care Unit, CHU de Montpellier, Montpellier, France; 10 Medical Intensive Care Unit, La Source Hospital, CHR Orleans, Orleans, France; 11 Medical Intensive Care Unit, Hotel Dieu, CHU de Nantes, Nantes, France; 12 Intensive Care Unit, CHR de Metz-Thionville, Metz, France; 13 Department of Perioperative Medicine, CHU Clermont-Ferrand, Clermont-Ferrand, France; 14 Intensive Care Unit, Lyon Sud Medical Center, Lyon, France; 15 Intensive Care Unit, Centre Hospitalier Metropole-Savoie, Chambery, France; 16 Medical Intensive Care Unit, Hopital Charles Nicolle, Rouen, France; 17 Medical Intensive Care Unit, Avicenne University Hospital, Bobigny, France; 18 Intensive Care Unit, Roubaix Hospital, Roubaix, France; 19 Medical Intensive Care Unit, CHU de Grenoble Alpes, Grenoble, France; 20 Medical Intensive Care Unit, CHU Bichat, Paris, France; 21 Medical Intensive Care Unit, Gabriel-Montpied University Hospital, Clermont-Ferrand, France; 22 Medical Intensive Care Unit, CHU St-Antoine, Paris, France; 23 Department of Anesthesia and Critical Care, Necker Hospital, Paris, France; 24 Reanimation Des Detresses Respiratoires Et Infections Severes, Assistance Publique-Hopitaux de Marseille, Hopital Nord, Aix-Marseille Universite, Faculte de Medecine, Marseille, France; 25 Medical Intensive Care Unit, Andre Mignot Hospital, Versailles, France; 26 Intensive Care Unit, Centre Hospitalier Departemental Les Oudairies, La Roche Sur Yon, France; 27 Department of Intensive Care, Ghent University Hospital, Ghent, Belgium; 28 Service de Médecine Interne, Soins Intensifs & Urgences Oncologiques, Institut Jules Bordet, Bruxelles, Université Libre de Bruxelles (ULB), Brussels, France; 29 PhyMedExp, INSERM U-1046, CNRS, Montpellier University Hospital, 34295 Montpellier, France

## Correction to: Ann Intensive Care (2021) 11:17 https:1//doi.org/10.1186/s13613-021-00801-z

Following publication of the original article [1], the authors identified an error in Figure 2.

The correct Fig. 2 is given.

**Fig. 2:**
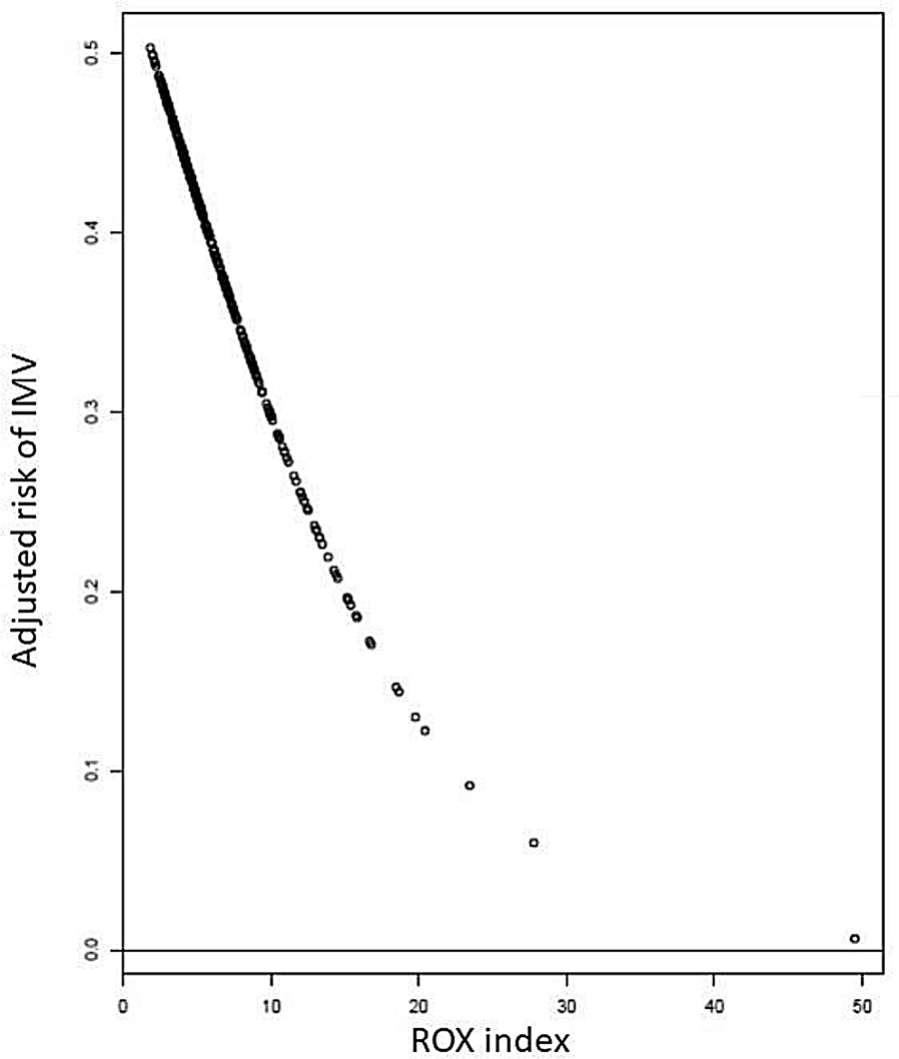
Probability of intubation according the ROX index
