# Supplementary material for: Performance of the ROX index to predict intubation in immunocompromised patients receiving high-flow nasal cannula for acute respiratory failure
Source: Ann Intensive Care. 2021 Jan 27;11:17. doi: 10.1186/s13613-021-00801-z (PMC7838224; doi:10.1186/s13613-021-00801-z)

ROX index to predict invasive mechanical ventilation in immunocompromised patients with acute respiratory failure?

Virginie Lemiale, MD^(1)^, Guillaume Dumas, MD^(1)^, Alexandre Demoule, MD, PhD^(2)^, Frederic Pène, MD, PhD^(3)^; Achille Kouatchet, MD^(4)^; Magali Bisbal, MD^(5)^; Saad Nseir, MD, PhD^(6)^; Laurent Argaud, MD, PhD^(7)^; Loay Kontar, MD^(8)^; Kada Klouche, MD, PhD^(9)^; Francois Barbier, MD, PhD^(10)^; Amelie Seguin, MD^(11)^; Guillaume Louis, MD^(12)^; Jean-Michel Constantin, MD, PhD^(13)^; Julien Mayaux, MD^(2)^; Florent Wallet, MD^(14)^; Vincent Peigne, MD^(15)^; Christophe Girault, MD^(16)^; Johanna Oziel, MD^(17)^; Martine Nyunga, MD^(18)^; Nicolas Terzi, MD, PhD^(19)^; Lila Bouadma, MD, PhD^(20)^; Alexandre Lautrette, MD, PhD^(21)^; Naike Bige, MD, PhD^(22)^; Jean-Herle Raphalen, MD^(23)^; Laurent Papazian, MD, PhD^(24)^, Fabrice Bruneel, MD^(25)^; Christine Lebert, MD^(26)^; Dominique Benoit, MD, PhD^(27)^; Anne-Pascale Meert, MD, PhD^(28)^; Samir Jaber, MD, PhD^(29)^; Djamel Mokart, MD, PhD ^(5)^, Michael Darmon, MD, PhD^(1)^; Elie Azoulay, MD, PhD^(1)^. The Groupe de Recherche en Reanimation Respiratoire du patient d’Onco-Hématologie (GRRR-OH)

**Additional material**

Figures and Tables legends

figure S1: ROC curve for ROX index 6 hours after HFNC onset

Area under the curve (AUC) : 0.623 (0.557-0.689)

Figure S2 : Probability of intubation according to the multivariable model including ROX index.

Figure S3: ROC curve of adjusted ROX index 6 hours after HFNC onset

Area under the curve (AUC) : 0.663 (0.596-0.729).

Figure S4: Comparison between ROC curves of adjusted ROX index and modified model without ROX index

ROC curve for adjusted ROX index 6 h after HFNC onset

ROC curve for modified model without ROX index

Likelihood ratio test p=0.002

Figure S5: Probability of intubation according to ROX index quartile.

For each ROX index quartile the probability of intubation was calculated including IC 95%.

Figure S1


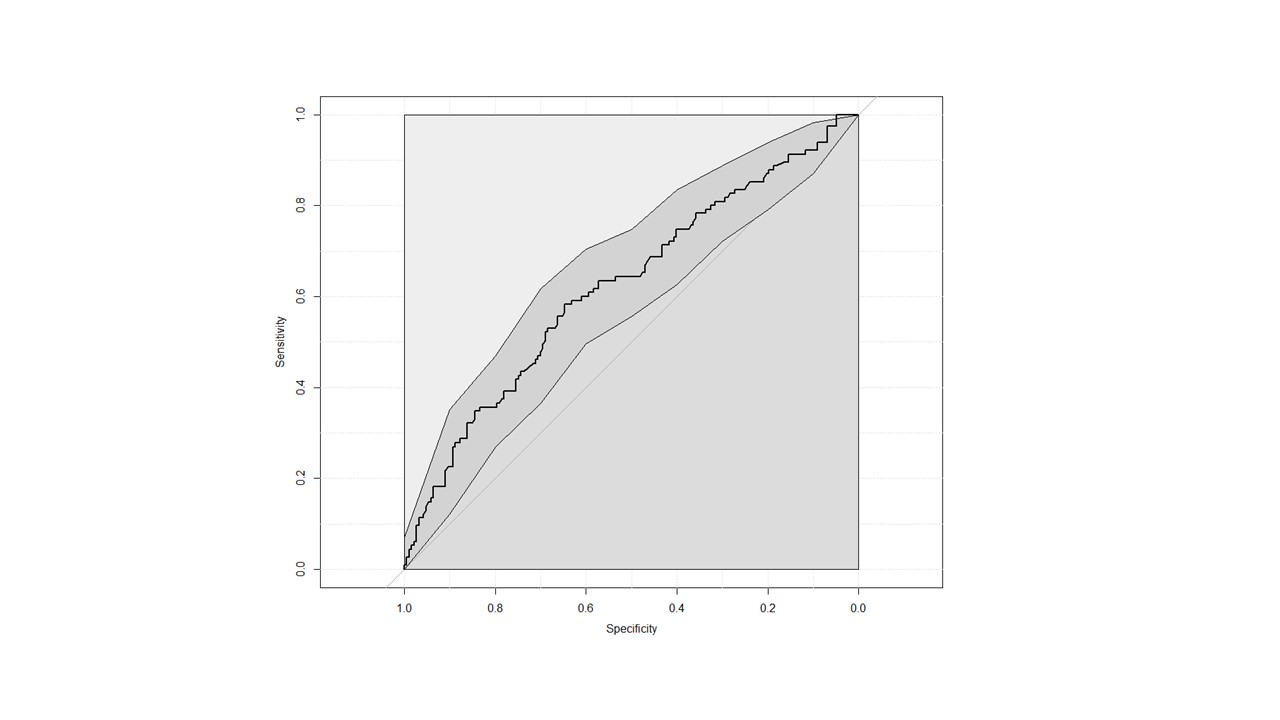


Figure S2


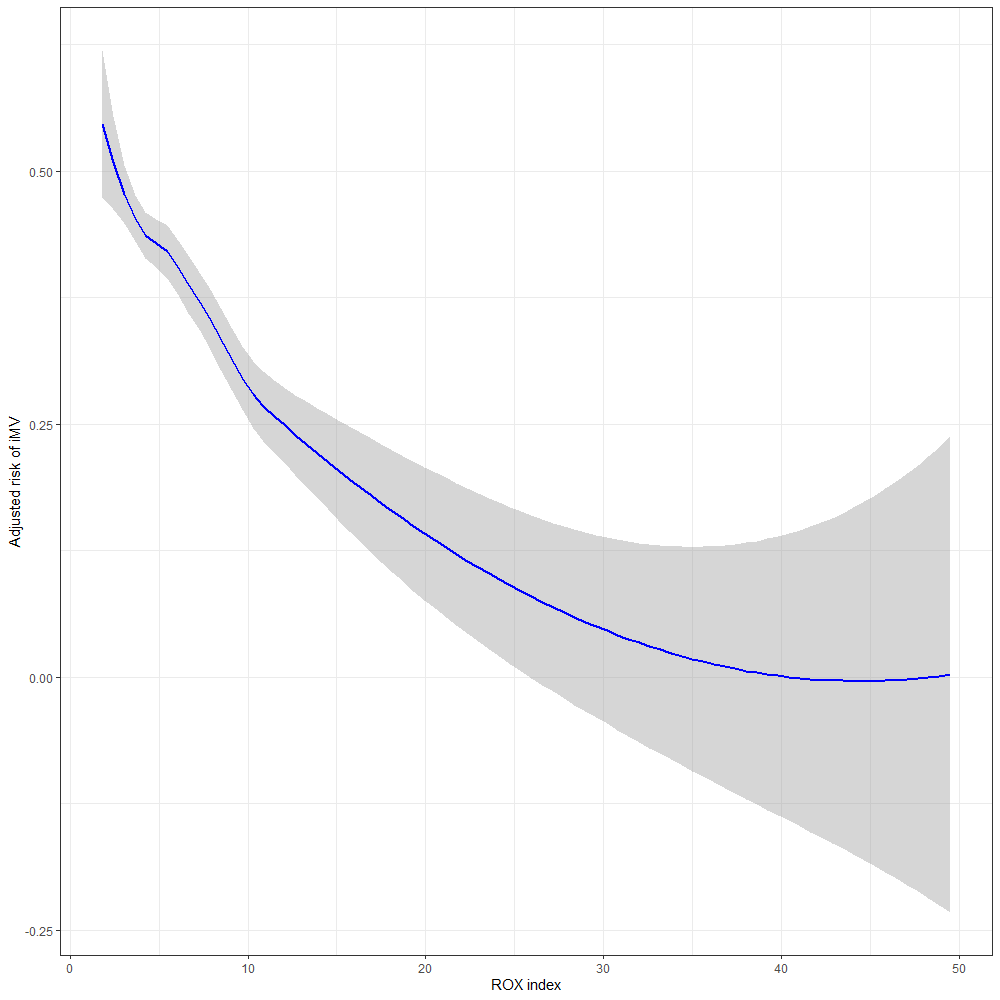


Adjusted ROX index

Figure S3


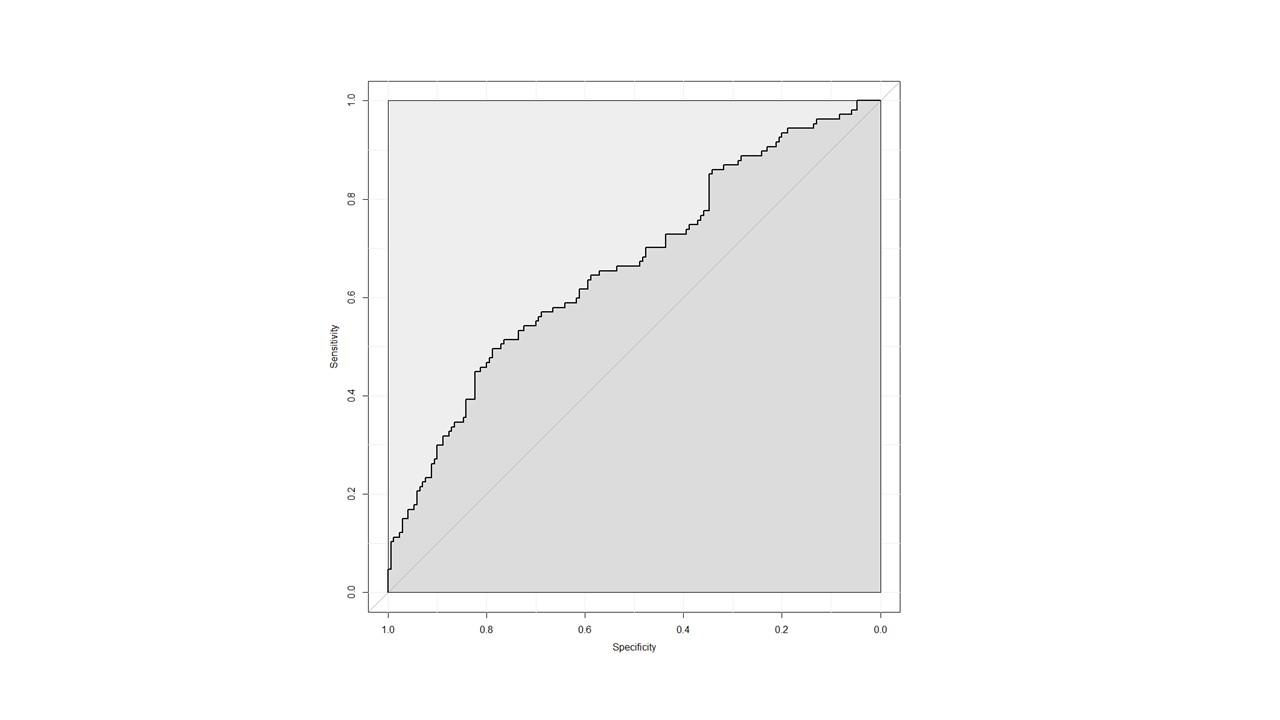


Figure S4


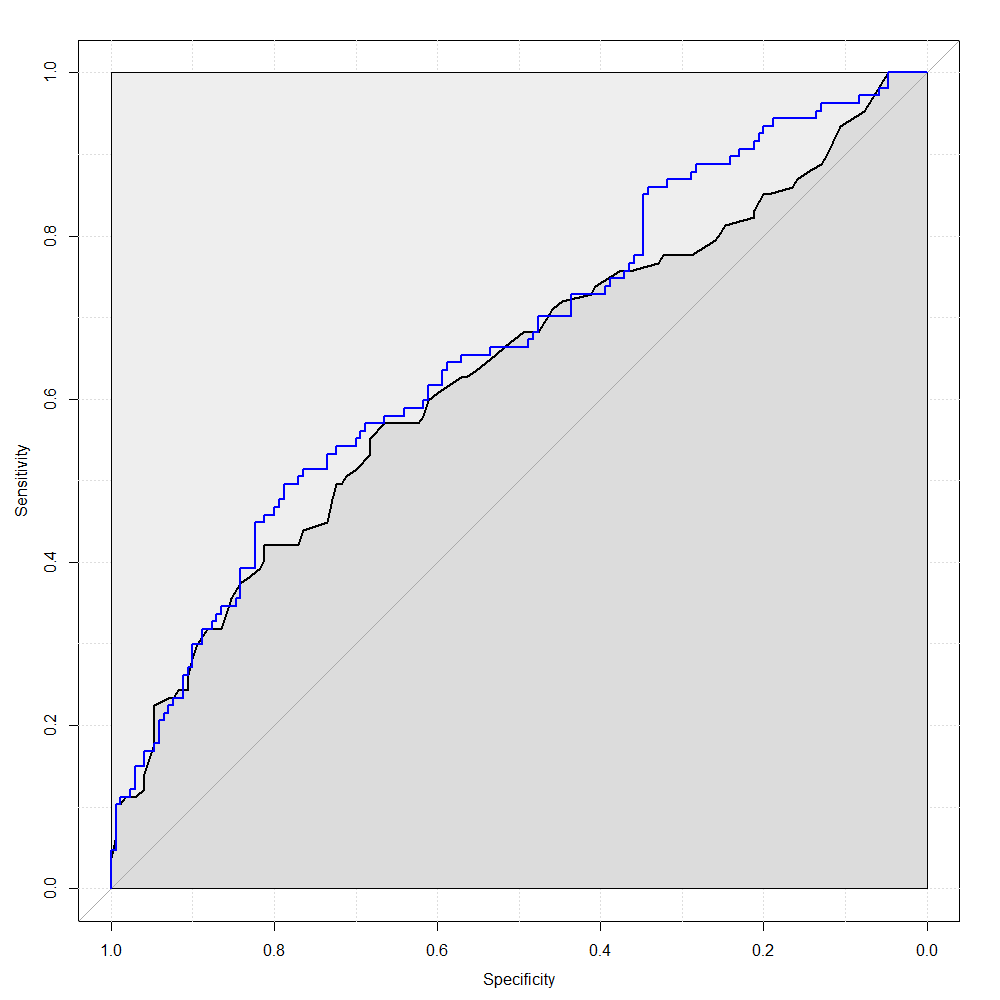


Figure S5


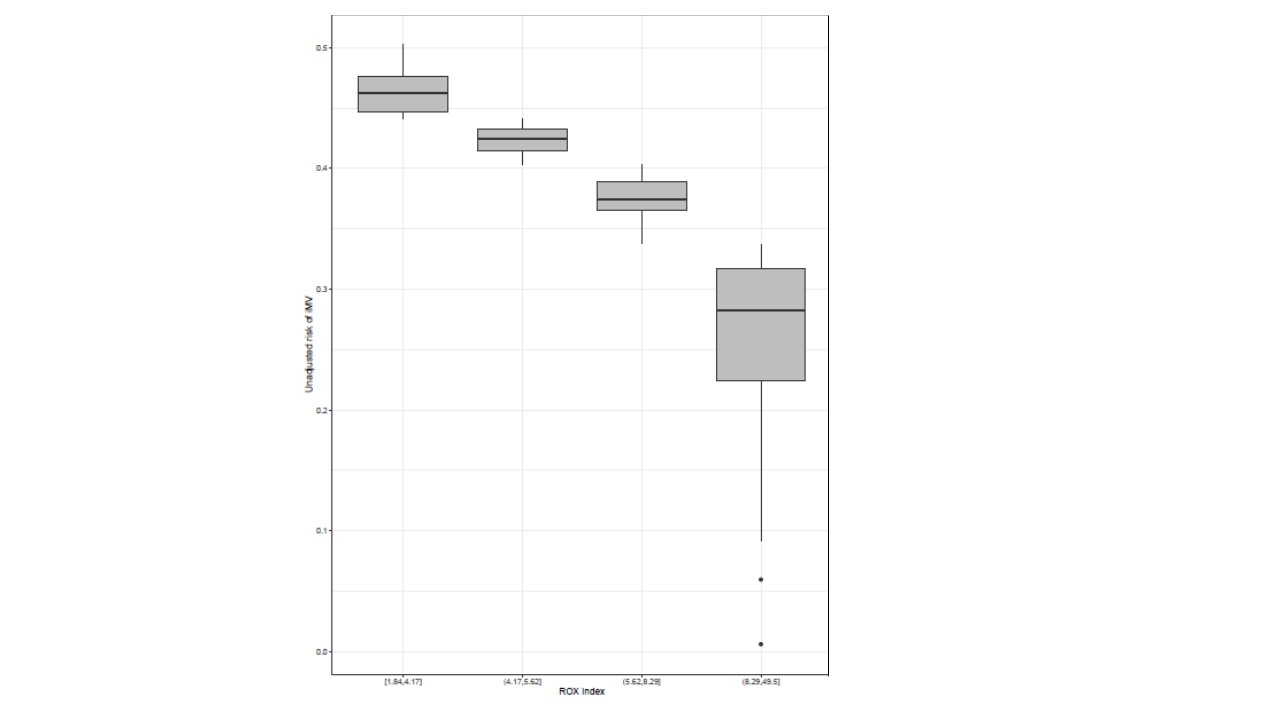

Supplement: Supplementary file 1 — Additional file 1: Figure S1. ROC curve for ROX index 6 hours after HFNC onset. Figure S2. Probability of intubation according to the multivariable model including ROX index. Figure S3. ROC curve of adjusted ROX index 6 hours after HFNC onset. Figure S4. Comparison between ROC curves of adjusted ROX index and modified model without ROX index. Figure S5. Probability of intubation according to ROX index quartile. [file 13613_2021_801_MOESM1_ESM.docx]
